# Nutrition regulation of male accessory gland growth and maturation in *Tribolium castaneum*

**DOI:** 10.1038/srep10567

**Published:** 2015-06-02

**Authors:** Jingjing xu, Ashlee L Anciro, Subba Reddy Palli

**Affiliations:** 1 Department of Entomology, college of Agriculture, University of Kentucky, Lexington, KY 40546, USA

## Abstract

Insulin/IGF-1 signaling (IIS) pathway is known to control growth, development and reproduction. Insulin-like peptide mediated body size plasticity in *Drosophila melanogaster* has been reported. Here, our studies showed that IIS pathway and nutrition regulate growth and maturation of the male accessory gland (MAG) in the red flour beetle, *Tribolium castaneum*. The size of MAG increased from day 1 to day 5 post-adult emergence (PAE). This increase in the size of MAG is contributed by an increase in cell size, but not cell number. The growth of MAG was impaired after double-stranded RNA (dsRNA)-mediated knockdown in the expression of genes coding for ILP3, InR, Chico, PI3k, AKT, and GATA1 involved in IIS pathway. Interestingly, starvation showed similar effects on the growth and maturation of MAG. The phenotypes observed in animals where IIS signaling pathway genes were knocked down are similar to the phenotypes observed after starving beetles for 5 days PAE. These data suggest that nutrition signals working through IIS pathway regulate maturation of MAG by promoting the growth of MAG cells.

Reproduction has never been independent of nutrition in animals including insects and mammals^1^,^2^,^3^,^4^,^5^,^6^. While there has been a great deal of research conducted on nutrition regulation of female reproduction, very little is known on how nutrition influences male reproductive traits.

Nutritional effect on male reproductive performance has been shown in both invertebrates and vertebrates. In *Drosophila melanogaster*, the males fed with diets containing different quantities of yeast showed no change in latency to first mating, mating duration and egg production. But males fed with diets containing different nutrient levels showed significant differences in mating receptivity with non-virgin females; males kept on intermediate nutrition fathered the most offspring^7^. In mammals, changes in nutrition levels of mature rams and goat bucks lead to profound responses in testicular size and therefore the rate of production of spermatozoa^8^. But in insects, the significant effects of nutrition on reproductive traits are mainly due to the changes in male accessory gland proteins (ACP) which are also referred to as seminal fluid proteins^9^. Importantly, the ACPs are transferred to the female along with the sperm during mating. These proteins facilitate chemical communication and mediate interplay between male and female^10^,^11^. ACPs are also shown to function in sperm movement, storage and protection^12^, induction of ovulation and oviposition in the female^9^,^13^ in a variety of insects. But how the nutritional cues influence the production of ACPs and their function remains largely unknown.

Several hormones including androgens are known to cause significant effects on the prostate development and physiology in humans^14^, other than the direct effect of endocrine factors on the reproduction, the metabolic defects such as the diabetes and obesity caused by endocrine factors often result in unsuccessful reproduction^15^,^16^,^17^. Recent studies also showed that the sertoli cells of the testis secrete insulin in diabetic mice, but the testis insulin does not appear to be critical for male fertility. Exogenously applied insulin rescues impaired spermatogenesis and consequently restores male fertility via the hypothalamic-pituitary-gonadal axis, and thus, normalizes levels of luteinizing hormone and testosterone in diabetic mice^18^. In male insects, reduced JH levels or mutations to JH receptor Methoprene-tolerant affect male reproductive fitness as evidenced by a less vigor in mating behavior, poor sperm transfer, low egg and progeny production by females in both *Tribolium castaneum* and *D. melanogaster*^19^,^20^.

Therefore, endocrine factors play conserved roles in all the animals from insect to mammals to support a successful reproductive process. However, little is known about how endocrine factors promote male reproduction during the MAG/prostate maturation.

In the previous study, we showed that nutrition and Insulin/IGF-1 (IIS) signaling pathway are required for vitellogenin gene expression in the red flour beetle, *T. castaneum*^21^, suggesting IIS function in *T. castaneum* is very much conserved with that in *D. melanogaster* and mammals. Also, *T. castaneum* males show considerable phenotypic plasticity in mating and insemination success responding to the nutrition^22^. To study molecular mechanisms governing IIS regulation of male reproduction, *T. castaneum* has been employed as a model species. RNA interference, confocal imaging, protein amount measurement and sperm production estimation were served to determine how nutritional signals working through IIS regulate male reproduction.

## Results

### Nutrition promotes male reproduction through MAG maturation

**Figure 1:**
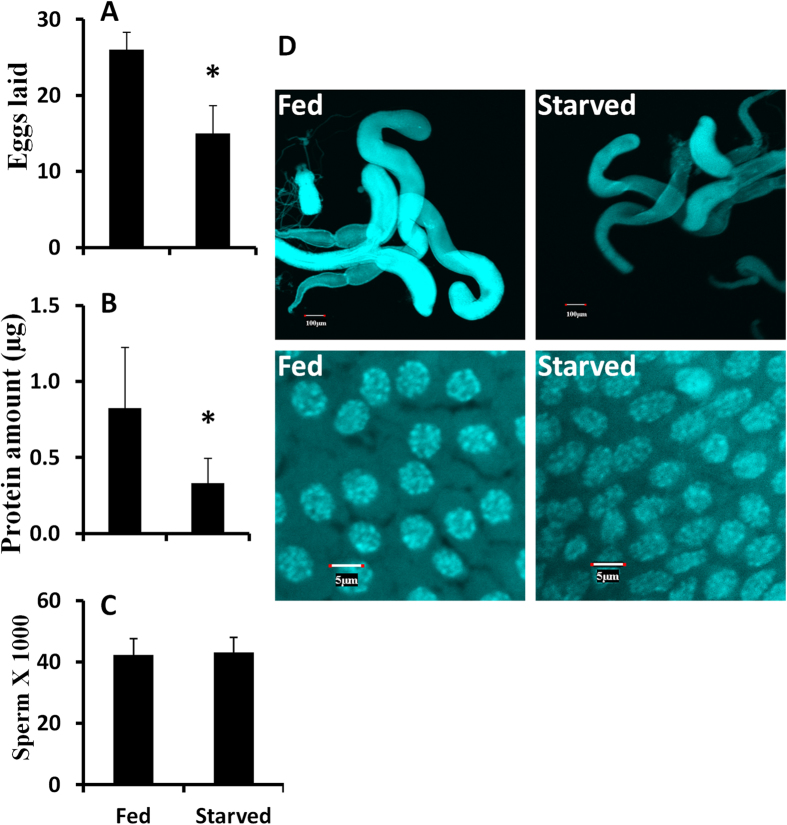
Effect of nutrition on male reproduction. **A**: Females mated with 5-day-old fed or starved males for a day, then the females and males were separated and the eggs laid were counted on 7^th^ day after mating. Shown are Mean + SD, n = 10. **B**: Male beetles were fed or starved for 5 days post adult emergence, then the total protein in seminal vesicle was determined by Bradford reagent for both fed and starved male beetles on day 5. Each treatment included five beetles. Shown are Mean + SD, n = 10. **C**: Same treatment as Fig. 1B except that the total sperm were counted for both fed and starved male beetles on day 5. Shown are Mean + SD, n = 10. **D**: Same treatment as Fig. 1B, except that the MAG and cell size in fed and starved male beetles on day 5 are shown PAE.

Our previous studies showed that nutrition is required for female reproduction. To study influence of nutrition on male reproduction, newly emerged males were separated into two groups, either starved from adult emergence or fed continuously for 5 days before they were mated with 5 day-old virgin females for one day. Both starved and fed males were placed in the same incubator set at 40% humidity based on our previous report^23^. Then the mated females were transferred to new cups and the eggs laid over a week period were collected. The females mated with the starved males laid 40% fewer eggs than did the females mated with fed males (Fig. 1A). A significantly less amount of total ACP was detected in the MAG of starved males when compared to that in normal males (Fig. 1B). In contrast, a similar number of sperm was detected in the starved males and the fed males (Fig. 1C). Therefore, the decrease in egg laying contributed by males is mostly due to the differences in the amount of male accessory gland proteins, but not due to difference in the number of sperm produced. Interestingly, the MAG in the fed beetles showed a larger size when compared to the MAG in starved males. The larger size of MAG is mainly due to an increase in the size of tubular accessory glands (TAG) and bigger cell size of the TAG (Fig. 1D). Taken together, these data showed that nutrition regulates TAG cell growth and maturation.

### ILP3 mediates nutritional regulation of MAG maturation

**Figure 2:**
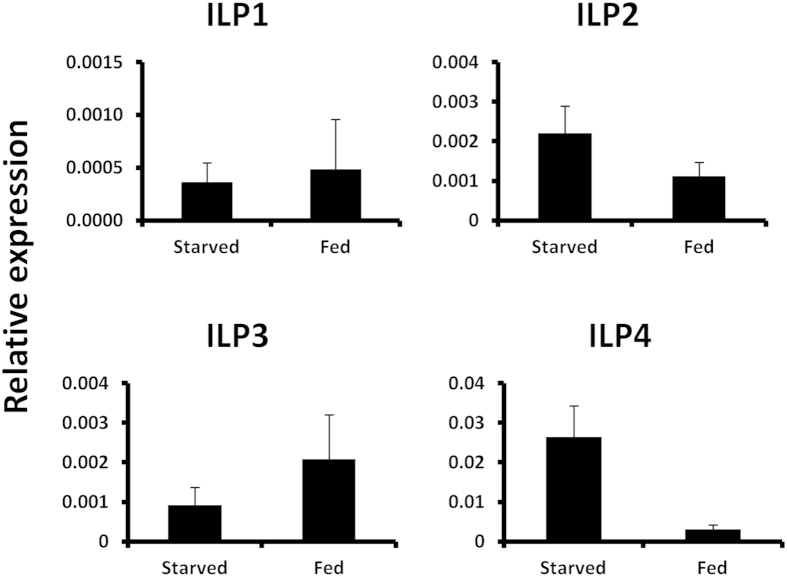
mRNA levels of ILPs in MAG dissected from fed and starved male beetles on day 5 after adult emergence. mRNA levels of all four ILPs in MAG dissected from fed and starved male beetles on day 5 PAE.

**Figure 3:**
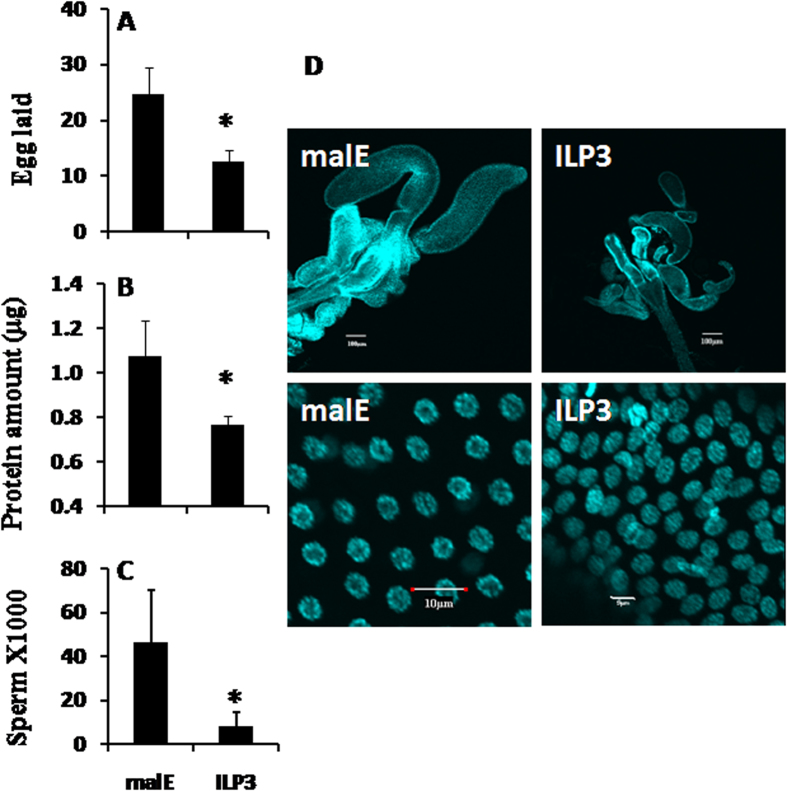
Effect of ILP3 knockdown on male reproduction. **A**: Eggs laid by females (from day 5 to day 12) mated with day 5 males injected with malE or ILP3 dsRNA on the day3 of pupal stage. Shown are Mean + SD, n = 10. **B**: The same treatment as Fig. 3A, except that the total protein levels in MAG were determined by Bradford reagent for both malE and ILP3 RNAi beetles on day 5 PAE. The MAGs were dissected on day 5 PAE, five MAGs were crushed in PBS solution; five replicates were performed for each treatment. Shown are Mean + SD, n = 5. **C**: The same treatment as Fig. 3A, except that the total sperm number was counted in malE and ILP3 RNAi beetles on day 5. The day 5 males injected with malE or ILP3 dsRNA were dissected, only the seminal vesicles were gently and completely removed and saved in a 1.5 ml tube. Dissection needle was used to crush and stain with DAPI. Shown are Mean + SD, n = 10. **D**: The same treatment as Fig. 3A, the MAG size and cell size in malE and ILP3 RNAi beetles on day 5 PAE are shown.

Out of four insulin-like peptides (ILPs) identified in *T. castaneum*^21^,^24^, higher levels of ILP3 mRNA were detected in the MAG of fed beetles when compared to its levels in MAG dissected from starved beetles (Fig. 2). In contrast, lower levels of ILP2 and ILP4 mRNA were detected in the MAG dissected from fed beetles when compared to their levels in MAG dissected from starved beetles (Fig. 2). Since nutrition positively regulates MAG maturation, thereby promoting male reproduction and also ILP3 mediates Vg expression by responding to nutritional signals in female reproduction^21^, ILP3 was chosen for further analysis. ILP3 dsRNA was injected into pupae and the male beetles emerged from these pupae were examined. When ILP3 RNAi males were mated with virgin females, the females produced less progeny when compared to the females mated with control males (Fig. 3A, *P* < 0.05). The ILP3 RNAi males produced a less amount of ACP proteins and less sperm than did the malE beetles (Fig. 3B,C). Similarly, a reduction in TAG size and cell size was observed in ILP3 RNAi beetles (Fig. 3D).

### Cell growth and TAG maturation

**Figure 4:**
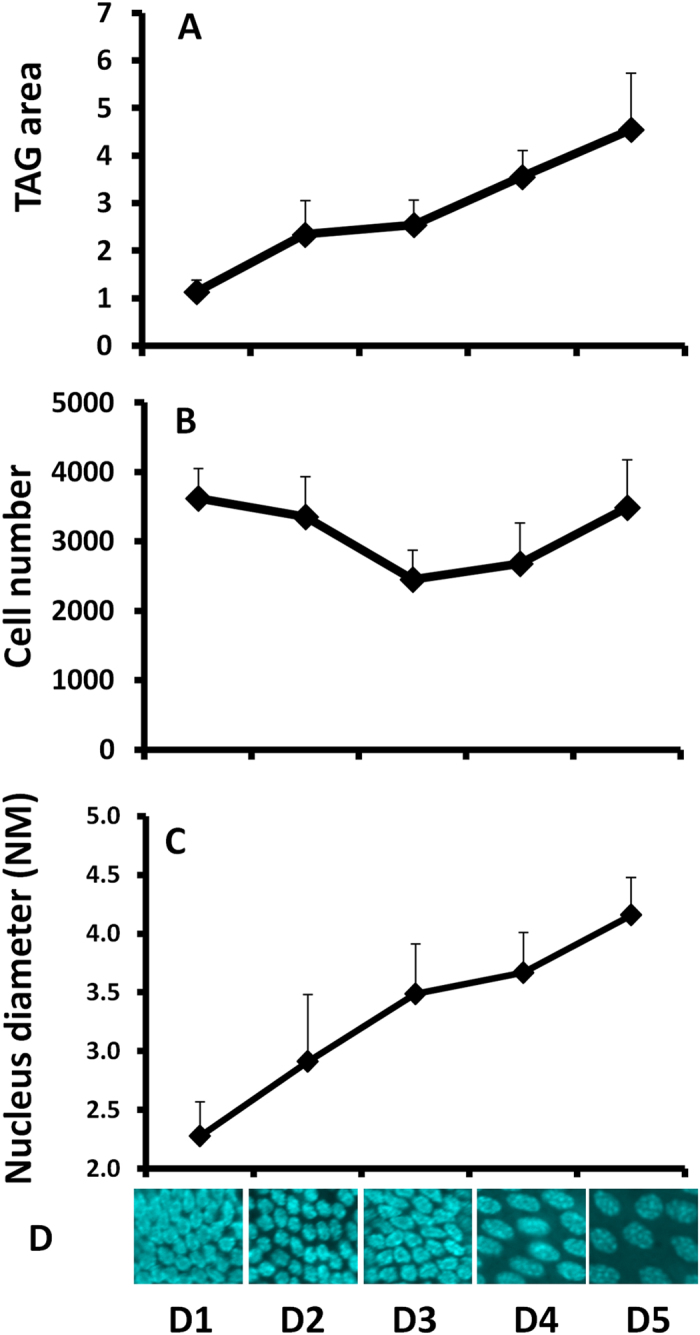
Cell growth in MAG from day 1 to day 5 after adult emergence. **A**: The cell number in MAG from day 1 to day 5, MAGs were dissected from day 1 to day 5 and stained with DAPI. Pictures were taken under a confocal microscope. Cell number was also counted. **B**: The TAG area from Day 1 to Day 5. **C**: The cell size from day 1 to day 5.

To answer the question how nutrition and ILP3 signaling pathway affect the development of MAG, We first characterized TAG growth during MAG maturation to determine whether an increase in TAG size is due to an increase in number or size of cells or both^19^. The size of the TAG increased by five times from day 1 to day 5 PAE (Fig. 4A), each pair of TAG contains around 3500 cells (Fig. 4B), and these cells showed a gradual increase in their size including nucleus and cytoplasm from day 1 to day 5 (Fig. 4C,D), The cell diameter increased almost by 2-fold from day 1 to day 5 (Fig. 4C). But the cell number did not show a significant change from day 1 to day 5.

### Insulin signaling pathway is involved in TAG maturation

**Figure 5:**
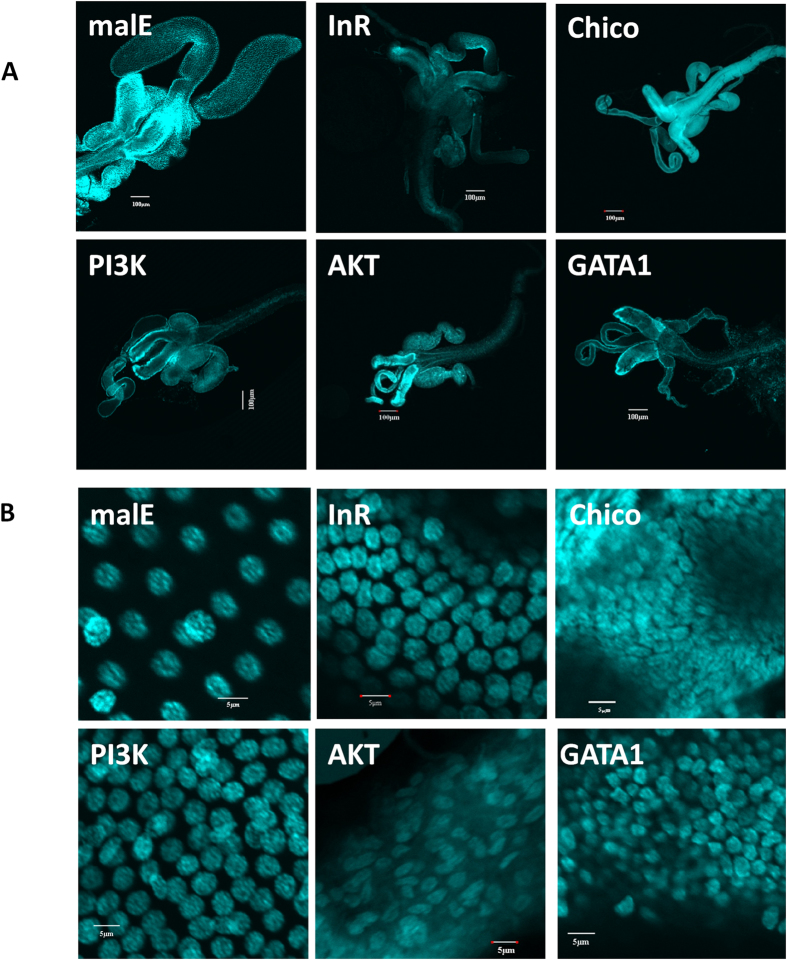
IIS pathway is involved in MAG maturation by promoting cell growth. **A**: The decrease in TAG size in an impaired IIS pathway. Shown is the decreased size of TAG in male beetles injected with InR, IIP3, Chico, Pi3k, AKT, GATA1 dsRNA. **B**: The same treatment as in Fig. 5A, shown is cell size decrease caused by an impaired IIS pathway.

To study the function of IIS pathway in MAG maturation via promoting cell growth, the genes involved in IIS pathway including insulin receptor (InR), insulin receptor substrate (Chico), phosphatidylinositol 3-kinase (PI3K), AKT and GATA1 were knocked down by injecting dsRNA targeting these genes. Strikingly, all males injected with dsRNA targeting IIS pathway genes showed a smaller TAG (Fig. 5A). The extent of reduction in TAG size varied depending on the IIS pathway gene silenced. Interestingly, knockdown of Chico or AKT caused a reduction in nuclei size when compared to the malE group. Knockdown in ILP3, InR, PI3K and GATA1 caused a reduction in TAG size due to a reduction in volume of cytoplasm but the size of nuclei did not change significantly (Fig. 5B, Table 1). The most severe effect was observed in AKT RNAi animals, the growth of TAG were blocked completely (Fig. 5B) suggesting that AKT might be an autonomous factor that plays a critical role in regulation of TAG growth and maturation.

### IIS pathway especially AKT are required for production of ACP

**Figure 6:**
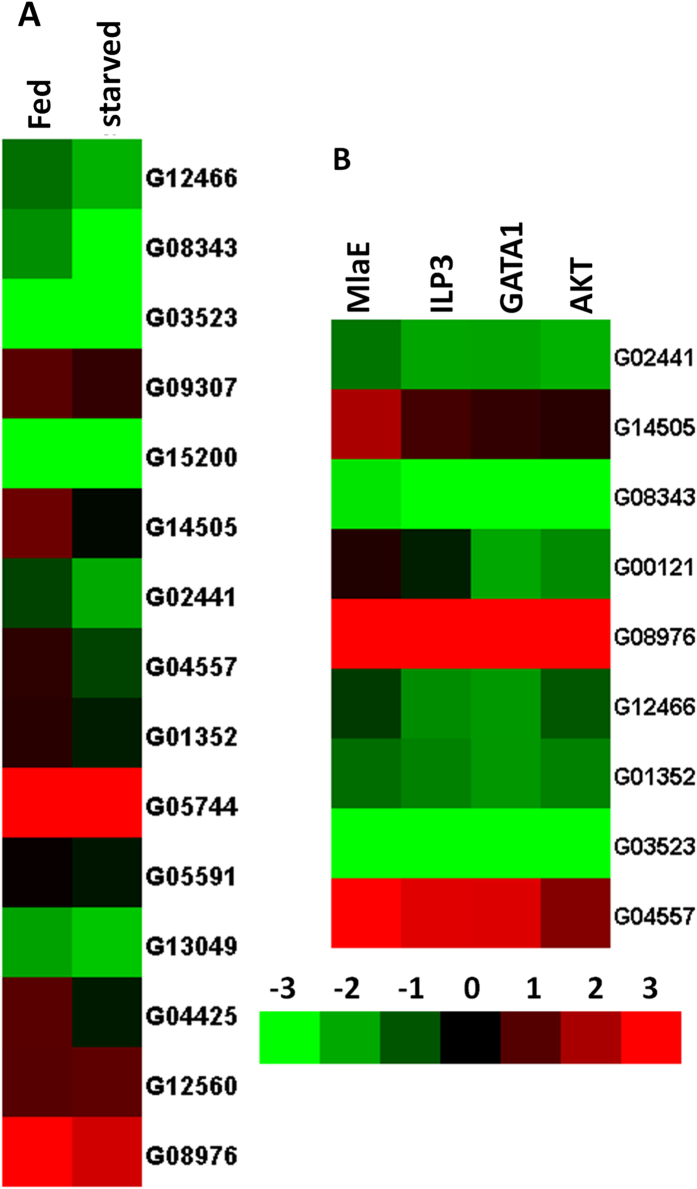
The Acp gene expression is regulated by nutrition and IIS pathway. **A**: The relative mRNA levels of nine Acp genes in MAG dissected from fed or starved male beetles on day 5 PAE. Total RNA was isolated from the MAG dissected from fed and starved beetles on day 5. Ten MAGs were used in each sample, four replicates were done for both fed and starved beetles. The total RNA was converted to cDNA, and the relative mRNA levels of nine ACPs were determined by qRT-PCR using RP49 as a control. The mean normalized values are displayed using Cluster 3.0 program. **B**: The relative mRNA expression of nine Acp genes in MAG dissected from beetles injected with malE, ILP3, AKT or GATA1 dsRNA. Day 3 pupa were injected with dsRNA, MAG were dissected on day 5 post adult emergence, Total RNA was isolated from the MAGs were dissected from fed or starved beetles. Ten MAGs were used for each sample, four replicates were done for fed and starved beetles. The total RNA was converted to cDNA, and the relative mRNA levels of nine ACP were determined by qRT-PCR using RP49 as a control. The mean normalized values are displayed using Cluster 3.0 program.

Nutrition-sensitive ACPs were selected by comparing mRNA levels of genes coding for ACPs between starved and fed beetles. We tested 15 genes highly expressed in the MAG as shown in our previous study^19^,^25^. Out of these 15 genes, nine genes including G08976, G03523, G14505, G04557, G02441, G12466, G08343, G00121 and G01352 showed significantly higher mRNA levels in the fed beetles than in the starved beetles (Fig. 6A). To determine the role of IIS in regulating the expression of these nutrition sensitive Acp genes, ILP3, AKT or GATA1 dsRNAs were injected into newly emerged adult males and the expression of all nine Acp genes were determined on day-5 PAE by qRT PCR. The mRNA levels of all nine Acp genes tested were lower in ILP3, AKT and GATA1 dsRNA injected beetles than in control beetles injected with *malE* dsRNA (Fig. 6B). These data suggest that IIS pathway plays an important role in regulation of production of these ACPs.

## Discussion

### AKT/GATA1 regulate cell growth in MAG

The main contribution of this study is that nutrition and IIS pathway regulate growth and maturation of MAG as well as production of ACP by promoting an increase in cell size but not number. It appears that the cell number in MAG is fixed after pupal-adult metamorphosis. After the newly emerged adults start feeding, the size in MAG increases due to an increase in cell size and this increase observed in starved beetles is lower when compared to the fed beetles suggesting that growth and maturation of MAG are controlled by at least two factors including intrinsic factor and nutritional signals. This intrinsic factor may include AKT. AKT appear to control MAG growth and maturation in a cell autonomous manner. PI3K/AKT regulatory pathways control cell size in MAG, which is consistent with the previous study in *Drosophila*, insulin/PI3K pathway coordinates cellular metabolism with nutritional conditions^26^. Knockdown of AKT during the late pupal stage completely blocked the development of TAG by arresting the cell growth in TAG (Fig. 5), which is the major gland responding to nutrition^19^. In *Drosophila* AKT appears to stimulate intracellular pathways that specifically regulate cell and compartment size independently of cell proliferation *in vivo*^27^, and the mechanisms involved include phosphorylating Tsc2^28^,^29^ and decrease in apoptosis^30^.

In the current study GATA1 has been identified as the terminal transcription factor involved in regulation of MAG growth and maturation. Out of five GATA genes tested, knockdown of GATA1 showed the same phenotypes as knockdown of other IIS pathway genes. To our knowledge, this is the first time to report on GATA1 as the transcription factor involved in IIS regulation of male reproduction. Interestingly, there is no difference in GATA1 mRNA levels between MAG isolated from fed and starved animals (Fig. S1) suggesting that IIS pathway may control GATA1 through post translational modification. Indeed, the role of GATA1 has been best studied in cell differentiation. In maturation of erythroid cells, GATA1 regulates proliferation, survival, and differentiation through the PI3k/AKT signaling pathway^31^. AKT phosphorylates GATA-1 in erythroid cells^31^ and K562 cells^32^ resulting in an increase in its DNA-binding affinity and enhanced transcriptional activity. Not only phosphorylation but also acetylation could alter interactions between transcription factors and DNA^33^. In female reproduction, Foxo was identified as the terminal transcription factor negatively regulating vitellogenin gene expression by binding to its promoter^21^, but no significant decrease in MAG size or cell size of MAG was observed when compared to the control group when Foxo was knocked down in males of *T. castaneum* (Fig. S2). Taken together, these studies showed that while Foxo is terminal transcription factor in regulation of vitellogenesis in females, GATA1 is the terminal transcription factor in regulation of MAG maturation in males of *T. castaneum*.

### Nutrition signals regulate cell growth in MAG through IIS signaling pathway

Nutritional signals play an important role to promote cell growth in MAG. The organ growth and maturation in insects is determined by multiple factors. For example, growth and differentiation of wing imaginal discs are regulated by endocrine factors such as Juvenile hormone, 20-hydroxyecdysone, and insulin-like peptides as well as nutrition^34^,^35^,^36^. In the adult, the reproductive organs such as ovary and MAG increase in size in mature beetles after they start feeding soon after adult emergence. Our previous studies showed that nutrition signals working through IIS pathway regulate oocyte maturation in adult females and survival in males^21^,^23^,^37^. But not much is known about nutrition regulation of male reproduction. In the current study, lack of nutrients did not affect sperm production, but influenced the MAG growth. Therefore, MAG is a simple and excellent system to study nutritional effect on ACP production. Organ growth is a consequence of increase in cell number, cell size, or both^38^. In MAG, nutrition signals working through IIS pathway facilitates the growth of MAG cells in adults beginning at soon after emergence. IIS regulation of cell growth, cell proliferation in *D. melanogaster*^39^,^40^, in the mosquito^41^,^42^,^43^ and in mice^30^,^43^,^44^ have been reported. Out of four ILPs tested, only ILP3 expression responds to the nutritional levels suggesting that ILP3 may be involved in transduction of nutritional signals. Likely MAG has its own mechanism to sense nutritional status and activates the PI3K/AKT signaling pathway to promote cell growth. As reported in *D. melanogaster*, some co-factors responding to nutritional signals may induce/activate ILP3^45^. In vertebrates, male reproductive tract also could be a local source of IIS as shown in the seminal vesicle^46^ and in the cells of human prostate^47^. Therefore, MAG is a good system to study IIS pathway function in the peripheral tissues.

### Cell growth in MAG contributes to the ACP synthesis

Target of Rapamycin (TOR) signaling is the best candidate to link nutrition to IIS pathway involved in cell growth, because of its known role in sensing external nutritional signals to the intracellular metabolism^38^,^48^,^49^. TOR has also been shown to cross-talk with IIS pathway through AKT^50^,^51^. A little but significant decrease in the cell size was observed after knockdown of TOR. In contrast, marginal level of increase in cell size was observed in TAG dissected from 4EBP RNAi beetles (Fig. S3). However, the phenotypes observed after TOR and 4EBP RNAi were much milder compared to the phenotypes observed in animals injected with dsRNA targeting IIS pathway genes. These data suggest that TOR/4EBP may mediate protein translation, which occurs after TAG cells grow and mature^52^. As evidenced by an increase in cell growth accompanied by an increase in expression of nutrition regulated Acp genes from day 1 to day 5 PAE Once the cell increases in size most likely due to an increase in protein synthesis machinery, the production of proteins increases resulting in an increase of MAG size (Fig. 4).

The male accessory gland is a very good model organ to study IIS pathway because of its parallels with human prostate and significant cell growth response to nutrition. Studies on the conserved function of IIS signaling may lead to identification of novel targets of AKT such as GATA, which then could be used as the marker for cell growth various diseases such as prostate cancer.

## Methods

### *Tribolium castaneum* rearing and staging

Strain GA-1 of *T. castaneum* was reared on organic wheat flour fortified with 10% baker yeast at 30 ± 1 °C as described previously^37^. New adults were separated within 6 h post emergence and staged from that time.

### Total ACP proteins amount and sperm estimation

To determine total protein amount in the MAG, four replicates were used for each dsRNA treatment, for each replicate, ten pairs of MAG dissected from male beetles were gently crushed in the 50 μl PBS, then protein concentration was determined using Bradford reagent (Bio-Rad).

To estimate the sperm number in a single beetle, the seminal vesicles were dissected from day 5 male, crushed and stirred in 50 μl PBS solution containing 1% BSA. The sperm were stained with the nucleic acid dye acridine orange. After a series of dilutions, 10 μl of the sperm suspension was counted on the slide viewed under a microscope.

### RNA isolation, cDNA synthesis and dsRNA synthesis

Total RNA was isolated using the TRI reagent (Molecular Research Center Inc., Cincinnati, OH). The DNA was eliminated from the total RNA using DNase I (Ambion Inc., Austin, TX) and 2 μg of total RNA for each sample was used for cDNA synthesis. Genomic DNA template was used to amplify fragments of target genes and the PCR products were used for dsRNA synthesis. Genomic DNA was extracted from *T. castaneum* adults and purified using the DNeasy Tissue Kit (QIAGEN). All the primers used for dsRNA synthesis and real time PCR have been published in our previous papers^19^,^25^. The MEGA script RNAi Kit (Ambion Inc., Austin, TX) was employed for dsRNA synthesis. For annealing dsRNA, the reaction mixture was incubated at 75 °C for 5 min and cooled it to room temperature over a period of 60 min. After the treatment with DNase I, dsRNA was purified by phenol/chloroform extraction followed by ethanol precipitation and the dsRNA concentration was determined using the Nano Drop 2000 spectrophotometer (Thermo Fisher Scientific Inc., Waltham, MA).

### Microinjection, estimation of egg laying and qRT-PCR

The newly hatched male adults (within 6 h after emergence) or the day 4 pupae were anesthetized with ether vapor for 4–5 min and lined on a glass slide covered with two-sided tape. The dsRNA was injected into the dorsal side of the first or second abdominal segment using an injection needle pulled out from a glass capillary tube using a needle puller (Idaho technology). About 0.8–1 μg (0.1 μl) dsRNA was injected into each adult or pupa. The dsRNA prepared with 800 bp bacterial malE gene was used as a template for preparing control dsRNA. The dsRNA-injected beetles were removed from the slide and raised in whole-wheat flour at 30±1 °C.

DsRNA-injected males were mated with virgin females on the 5^th^ day after adult emergence. One RNAi adult male and one virgin female were placed in each cup containing flour. The eggs laid by each female were separated and counted on the seventh day after initiation of matting.

Relative mRNA levels of ACP dissected from dsRNA injected beetles were determined by qRT-PCR. cDNA prepared using RNA isolated from dsRNA injected male adults, primers designed based on ACP sequences and Step one plus Real-Time PCR system Thermal Cycling Block (Applied Biosystem, Foster city, CA ) were used to perform qRT-PCR. The primers for qRT-PCR were designed based on the sequence of genes coding for ACPs; regions that are outside the dsRNA target regions were used to design qRT-PCR primers. qRT-PCR reactions were performed using a common program as follows: initial incubation of 95 °C for 3 min was followed by 40 cycles of 95 °C for 10 s, 55 °C for 1 min. Standard curves were obtained using a 10-fold serial dilution of pooled cDNA. Quantitative mRNA measurements were performed in triplicate and normalized to an internal control of *T. castaneum* ribosomal protein 49 (RP49) mRNA.

### Imaging and cell number counting

Olympus FV1000 laser scanning confocal microscope was used for imaging and documentation. DAPI was excited using 405 nm laser line. Control of microscope as well as image acquisition and exportation as TIFF files were conducted using Olympus Fluoview software version 1.5. Optical sectioning was done and composite Z-stack image was used wherever necessary. Figures of micrographs were assembled using Photoshop 7.0. Cell number, size and area were estimated as described previously^53^.

### Statistical analysis

The one-way ANOVA was performed for analysis of egg produced by RNAi beetles and protein amount in MAG was analyzed using the Bonferroni test.

## Additional Information

**How to cite this article**: Xu, J. *et al.* Nutrition regulation of male accessory gland growth and maturation in *Tribolium castaneum*. *Sci. Rep.* **5**, 10567; doi: 10.1038/srep10567 (2015).

## Figures and Tables

**Table 1:** Cell diameter in MAG dissected from beetles injected with malE, InR, Chico, PI3K, AKT and GATA1 dsRNA

**Gene**	**malE**	**InR**	**Chico**	**PI3K**	**AKT**	**GATA1**
Nucleus size (nanometers, Mean+S.D.)	4.38 ± 0.45	3.53 ± 0.52	2.56 ± 0.12*	4.23 ± 0.26	1.68 ± 0.4*	2.92 ± 0.5
Number of adults tested	12	13	12	12	13	12

^*^denotes significantly different from control malE injected beetles
